# Rupture pressure values of cerebral arteries in the presence of unruptured intracranial aneurysm

**DOI:** 10.1038/s41598-022-13341-8

**Published:** 2022-06-18

**Authors:** Leszek Lombarski, Przemysław Kunert, Sylwia Tarka, Adam Piechna, Sławomir Kujawski, Andrzej Marchel

**Affiliations:** 1 Department of Neurosurgery, Medical University of Warsaw, Banacha St. 1a, 02-097 Warsaw, Poland; 2 Department of Forensic Medicine, Medical University of Warsaw, Oczki St. 1, 02-007 Warsaw, Poland; 3 Institute of Automatic Control and Robotics, Warsaw University of Technology, św. Andrzeja Boboli St. 8, 02-525 Warsaw, Poland; 4 Department of Exercise Physiology and Functional Anatomy, Collegium Medicum in Bydgoszcz, Nicolaus Copernicus University in Toruń, M. Skłodowskiej-Curie St. 9, 85-094 Bydgoszcz, Poland

**Keywords:** Neurology, Pathogenesis, Risk factors

## Abstract

Cerebral arteries (CAs) are prone to the saccular aneurysm formation. Since aneurysms may be considered as balloon-like dilations of the locally weakened arterial wall, it should be determined whether the presence of intracranial aneurysm is related to the generalized weakening of CAs. Among 184 consecutive forensic autopsies, eight brains with a single unruptured saccular aneurysm were identified. Aneurysms with adjacent CAs and specific CA segments were excised, namely: the anterior communicating artery complex, and bifurcations of the basilar artery, internal carotid arteries, and middle cerebral arteries. Then, aneurysm and CA specimens were subjected to pressure-inflation tests until rupture occurred at the arterial bifurcation or at the wall of the CA or aneurysm. The same protocol was applied to the control group composed of CAs excised from eight brains without aneurysm. No significant differences were noted between the experimental and control groups, depending on the mean rupture pressure (1054 vs. 1048 mmHg) and rupture site (bifurcation vs. wall) of the analyzed specimens. These findings indicate that the presence of unruptured saccular aneurysm is not related to generalized weakening of CAs among autopsy subjects. Moreover, the CA bifurcations do not represent regions of decreased wall strength.

## Introduction

Cerebral arteries (CAs) are particularly prone to aneurysm formation, with an incidence of 3.6–6% in the general population^1^. Furthermore, saccular intracranial aneurysm (sIA) rupture is the most prevalent cause of non-traumatic subarachnoid hemorrhage associated with a high mortality rate^2^. The aforementioned predilection of CAs to sIA development may result from differences in structure-related biomechanical properties of the arteries in diverse vascular beds.

CAs are characterized by the increased stiffness as compared to their extracranial counterparts^3^. This may be related to the paucity of elastin, which is mainly confined to the internal elastic lamina within the wall of CAs^4^. Additionally, progressive elastin degradation leads to further stiffness increase of CAs with age^5^. According to the literature data, CAs of patients harboring sIA may display particular morphological features, such as larger diameter of the MCA trunk or wider bifurcation angle of the MCA^6^ as well as more pronounced difference between diameters of the MCA main branches^7^. However, there is a paucity of data concerning the distinct biomechanical properties of CAs obtained from patients with sIA^8^. Since mechanisms of the initiation, growth, and rupture of sIAs comprise the interplay between physical and biological processes^9^, studies concerning the biomechanical properties of CAs may shed light on the uncertain pathogenesis of sIAs. In the present study, we aimed to compare rupture pressure values of specific CA segments harvested from autopsy subjects with and without unruptured sIA and determine whether presence of unruptured sIA is associated with generalized weakening of the wall of CAs.

## Materials and methods

### CA specimens

During 184 consecutive forensic autopsies, we analyzed brains of patients who died due to extracerebral reasons. Duration between the time of death and biomechanical tests did not exceed 36 h. All cadavers were stored at 4 °C before the autopsy. Single unruptured sIA was identified in eight brains (age 62 ± 4 years; 2 females). The control group consisted of eight brains without sIA, matched in terms of age and sex of the autopsy subjects (age 62 ± 7 years; 2 females). The sIAs with adjacent CAs were excised using a surgical microscope (Carl Zeiss OPMI pico S100, Germany). Then, specific CA segments characterized by increased risk of sIA formation were collected, including the anterior communicating artery (ACommA) with anterior cerebral arteries (ACAs), basilar artery (BA) bifurcation with posterior cerebral arteries (PCAs), and bifurcation of both internal carotid arteries (ICAs) and middle cerebral arteries (MCAs). Six corresponding CA segments from each autopsy subject were prepared for the tests (1 ACommA, 1 BA, 2 ICA and 2 MCA)—a total of 96 specimens were analyzed. All experiments were performed in accordance with relevant guidelines and regulations^10,11^. The Bioethics Commission at the Medical University of Warsaw was consulted and confirmed that our study did not require formal approval or consent.

### Measurements and pressure-inflation tests

**Figure 1:**
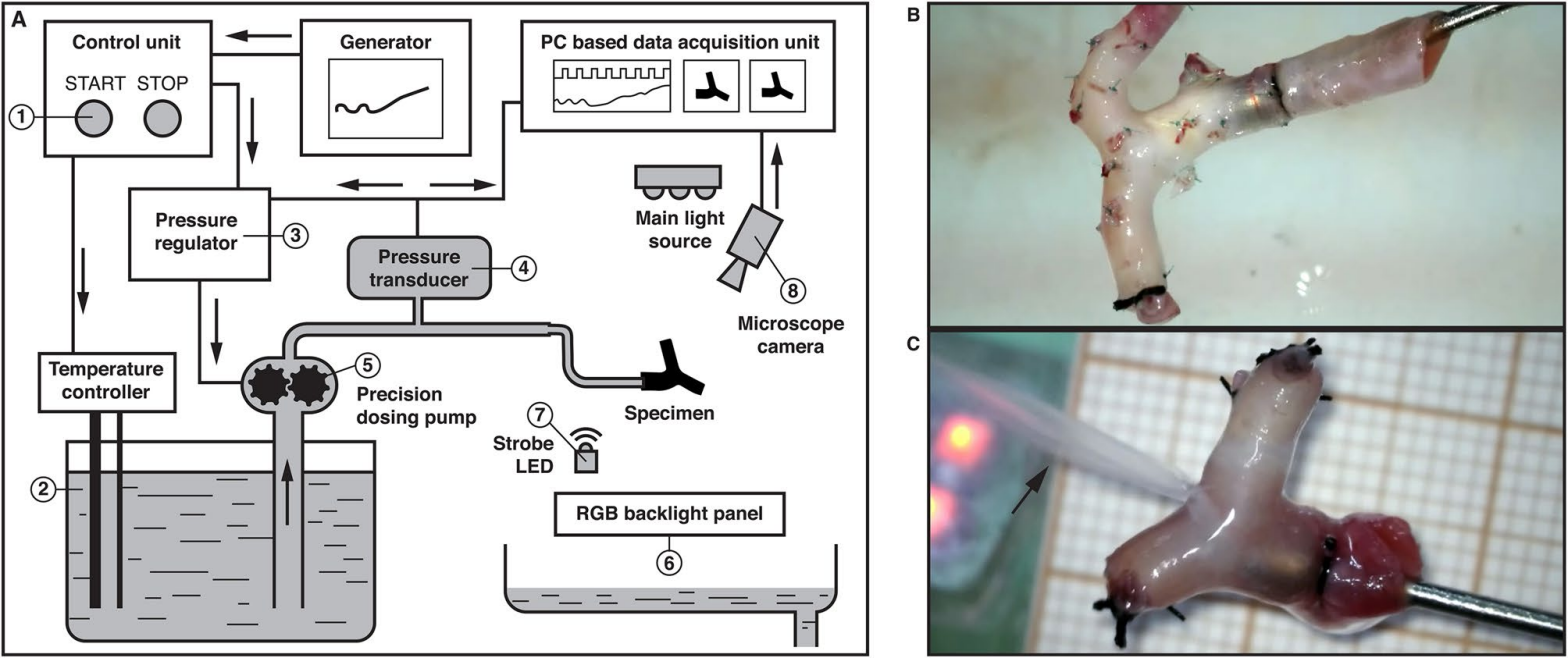
(**A**) A block diagram of a working area. Following the activation (1), temperature controller regulates the temperature of the 0.9% NaCl (2) maintaining its predefined value and sends a set point to pressure regulator (3). The feedback signal from the pressure transducer (4) regulator maintains proper pressure within analyzed specimen by controlling the precision dosing pump (5). Multicolor lights (6) provide optimal conditions for visual registration. LED diodes (7) were used to correlate the pressure with the image from the camera (8). (**B**) BA bifurcation specimen mounted on a flared tip cannula before the pressurization. Both opposite ends, i.e., PCAs, as well as perforating branches, are ligated with 4.0 and 7.0 silk sutures, respectively. (**C**) ICA bifurcation specimen at the time of rupture within the bifurcation region during pressure-inflation test; black arrow indicates the stream of 0.9% NaCl.

Biomechanical experiments of sIA and CA specimens were performed in a working area, which allowed for continuous measurement of intravascular pressure with simultaneous visual registration (Fig. 1A). All analyzed specimens were rinsed with 0.9% NaCl to remove the blood clots. Then, a flared tip cannula was inserted into the prepared specimen. The proximal end of the specimen was ligated with a surgical suture [4.0 silk suture] to ensure tight attachment to the cannula. To form a closed system, the opposite ends, as well as minor cortical and perforating arteries, were ligated using 4.0 and 7.0 silk sutures, respectively (Fig. 1B). Next, a precision dosing pump that ran 0.9% NaCl at 36 °C was activated. In the first stage, five preconditioning cycles were performed with gradually increasing–decreasing pressure ranging from 0 to 200 mmHg at a speed of 10 mmHg/s for muscle fiber relaxation. In the second stage, the diameters and lengths of the CA specimen were measured at a constant pressure of 100 mmHg to establish the approximate in vivo dimensions (for schematic representation of the performed measurements see Supplementary Fig. S1 online). To minimize the influence of the ligation on the intramural stress during pressure-inflation tests, the length of the prepared whole CA specimen was about four times of its diameter. Then, the specimen was subjected to quasi-static increasing pressure at a rate of 20 mmHg/s until the arterial bifurcation, or the wall of the CA or sIA, ruptured (Fig. 1C). The follow-up steering control system regulated the pump revolutions to provide a constant increase in pressure—the example of pressure recording obtained during preconditioning cycles and pressure-inflation test is presented in Supplementary Fig. S2 online. Differences between the aneurysm and non-aneurysm groups in terms of rupture pressure values of specific CA segments and rupture sites of pressurized specimens were analyzed. Furthermore, association of the obtained rupture pressure values with age of the autopsy subjects was evaluated for both groups separately.

### Statistical analysis

Statistical analyses were performed using the statistical package STATISTICA 13.1 (StatSoft, Inc.) and R environment. All continuous and ordinal variables were summarized as mean and standard deviation (SD). Percentages, numerators, and denominators were presented for categorical and binary variables. Student’s t-test for independent samples was used to examine differences between the two groups in continuous variables. Fisher's exact test was used to examine the qualitative variables. To examine differences between more than two groups, the Kruskal–Wallis ANOVA test was used. Regardless of the results of the main analysis, post-hoc analysis was conducted to compare each subgroup with other using the Dunn test with Benjamini and Hochberg p-value adjustment to control for the false discovery rate (FDR). Therefore, only p-values significant after correction for multiple comparisons (pFDR-corrected) are reported. Pearson’s correlation was used to measure the linear relationship between variables. For all calculations, the statistical significance level was set at α = 0.05.

## Results

### CAs rupture pressure

**Figure 2:**
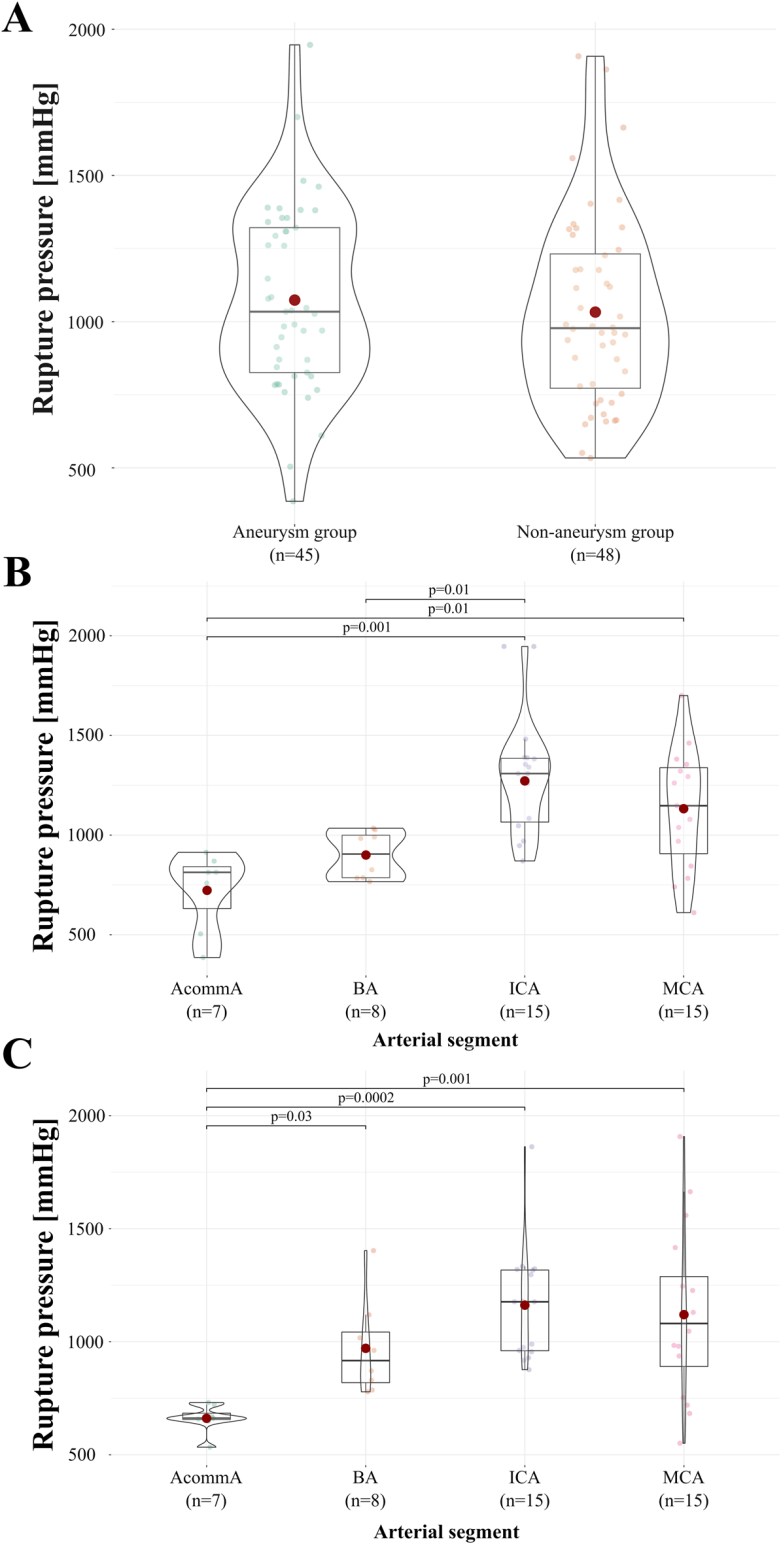
(**A**) Comparison of rupture pressure values of CAs between the aneurysm and non-aneurysm groups. (**B**) Comparison of rupture pressure values of specific CA segments in the aneurysm and (**C**) non-aneurysm group. Data represent violin plots, red dots indicate the arithmetic mean value, a horizontal black line inside the frame represents the median value, dots inside the graphs indicate the results of individual patients (dot positions along the abscissa are set to increase their visibility), and the shape of the graph indicates the distribution of values.

Table 1 presents detailed demographic data as well as rupture pressure values of analyzed aneurysms and CA segments. The mean rupture pressure of CAs in the control group was 1048 ± 323 mmHg, and the mean rupture pressure of CAs harvested from brains with single unruptured sIA was 1054 ± 289 mmHg. No significant differences were observed in rupture pressure values between groups (p = 0.54) (Fig. 2A). Since there were no significant differences in rupture pressures between right- and left-paired ICA and MCA in aneurysm (p = 0.26 and p = 0.95, respectively) and non-aneurysm (p = 0.76 and p = 0.38, respectively) group, both ICAs and both MCAs were analyzed as combined subgroups (see Supplementary Table S1 online). The average rupture pressures of ACommA were 723 ± 199 mmHg and 661 ± 60 mmHg; BA, 900 ± 119 mmHg and 971 ± 211 mmHg; ICA, 1272 ± 267 mmHg and 1162 ± 252 mmHg; MCA, 1132 ± 304 mmHg and 1120 ± 375 mmHg in the aneurysm and non-aneurysm groups, respectively. There were no significant differences in rupture pressure of ACommA (p = 0.42), BA (p = 0.42), ICA (p = 0.25), and MCA (p = 0.92) between the experimental and control groups (Table 2). Furthermore, there were significant differences in the mean rupture pressure values between specific CA segments in the aneurysm group (p = 0.0003). That is, as results of the post-hoc analysis in the aneurysm group has shown, the rupture pressure values of the ICA were significantly higher than those of the ACommA (p = 0.001) and BA (p = 0.01), as well as MCA compared to ACommA (p = 0.01) (Fig. 2B). Similarly, there were significant differences in the average rupture pressure values between the aforementioned CA segments in the non-aneurysm group (p = 0.0003). That is, as results of post-hoc analysis revealed, there was a significantly lower rupture pressure values of ACommA compared to the remaining CA segments: BA (p = 0.03), ICA (p = 0.0002), and MCA (p = 0.001, Fig. 2C).

**Table 1:** Demographic data, intracranial aneurysm location and rupture pressure values of aneurysms and particular CA segments.

Aneurysm	Age	Sex	Cause of death	Aneurysm location	Rupture pressure (mmHg)						
					Aneurysm	ACommA	BA	R ICA	L ICA	R MCA	L MCA
+	60	M	Acute myocardial infarction	R MCA bif		803	1021	1462	1323	1304	956
+	55	F	Pneumonia	ACommA	672	^a^	1014	1337	1921	1678	1277
+	60	M	Suicidal hanging	L MCA bif		749	971	1364	1372	1337	1443
+	63	F	Generalized cancer	L ICA bif		381	815	957	1292	1132	1245
+	67	M	Suicidal hanging	L MCA bif	588	498	774	934	859	603	^a^
+	58	M	Drowning	L ICA bif	1017	858	776	1033	^a^	833	773
+	69	M	Alcohol poisoning	ACommA		803	756	1070	1243	1065	730
+	61	M	Alcohol poisoning	L ICA bif		902	977	1292	1370	1025	1363
−	56	M	Suicidal hanging			722	769	1299	977	710	544
−	59	M	Myocarditis			663	776	917	1280	674	967
−	55	M	Acute myocardial infarction			713	1385	906	1838	1229	1100
−	65	F	Pulmonary embolism			655	819	1316	1303	925	1398
−	72	F	Acute myocardial infarction			650	860	1305	963	1211	971
−	70	M	Fall from height			527	949	1161	1164	1033	1115
−	52	M	Generalized cancer			641	1105	949	865	1642	1883
−	66	M	Acute myocardial infarction			653	1004	1161	943	743	1539

+ present, − absent, *M* male, *F* female, *R* right, *L* left, *ACommA* anterior communicating artery, *BA* basilar artery, *MCA bif* middle cerebral artery bifurcation, *ICA bif* internal carotid artery bifurcation.

^a^In 3 specimens the aneurysm ruptured, and these specimens were excluded from the analysis regarding the rupture pressure values of CAs.

**Table 2:** Mean rupture pressure of specific CA segments depending on the presence of unruptured sIA.

	Aneurysm group		Non-aneurysm group		p-value
	Rupture pressure		Rupture pressure		
	Mean	SD	Mean	SD	
ACommA (mmHg)	723	199	661	60	0.42
BA (mmHg)	900	119	971	211	0.42
ICA (mmHg)	1272	267	1162	252	0.25
MCA (mmHg)	1132	304	1120	375	0.92
Total (mmHg)	1074	315	1033	324	0.54

*ACommA* anterior communicating artery, *BA* basilar artery, *ICA* internal carotid artery, *MCA* middle cerebral artery, *SD* standard deviation.

### SIAs rupture pressure

Among the eight analyzed sIAs, two were located at the ACommA complex, three at the bifurcation of the ICA, and three at the bifurcation of the MCA. Rupture of the sIA wall was identified in three specimens: ACommA, ICA, and MCA aneurysm. The average sIA rupture pressure was 769 ± 230 mmHg. In the other five specimens, rupture occurred within the arterial wall.

### Rupture site—bifurcation region vs. wall of the straight portion of CA specimens

During the pressure-inflation tests, rupture predominantly occurred at the wall of the straight portion of CAs in 32/38 (84%) and in 34/40 (85%) of the analyzed specimens in the experimental and control groups, respectively. Specifically, in the aneurysm group, the rupture occurred at the bifurcation region in 6/15 (40%) of MCAs, while no rupture was observed in the bifurcation region of ICAs; meanwhile, in the non-aneurysm group, rupture at the bifurcation region was observed in 4/16 (25%) of MCAs and 2/16 (12.5%) of ICAs. No rupture was identified in the bifurcation region of the BA in either group. No significant differences were noted between groups regarding the rupture site (bifurcation region vs. wall of the straight portion) of BA (p = 1), ICA (p = 0.48), and MCA (p = 0.46) (Table 3). Moreover, there were no significant differences regarding rupture pressure values of the MCA (p = 0.89) depending on the rupture site in the group with sIA. Similarly, no significant differences were observed between the rupture pressure values of the ICA (p = 0.93) and MCA (p = 0.79) depending on the rupture site of the analyzed specimen in the control group.

**Table 3:** Rupture site of specific CA segments depending on the presence of unruptured sIA.

	Aneurysm group		Non-aneurysm group		p-value
	Rupture site		Rupture site		
	Bif	Wall	Bif	Wall	
BA	0	100%	0	100%	1
ICA	0	100%	12.5%	87.5%	0.48
MCA	40%	60%	25%	75%	0.46
Total	16%	84%	15%	85%	1

*ACommA* anterior communicating artery, *BA* basilar artery, *ICA* internal carotid artery, *MCA* middle cerebral artery, *Bif* bifurcation.

### Association of rupture pressure with the dimensions of CA specimens

The mean diameter and length of the prepared segments of particular CAs obtained at a constant pressure of 100 mmHg are presented in Table 4 (for raw data see Supplementary Tables S2–S7 online). Except of A1 diameter (p = 0.04) and A2 length (p = 0.003), there were no significant differences between the average diameter and length of particular CAs in aneurysm and non-aneurysm groups. We have observed significant positive correlations between rupture pressure and diameter in aneurysm (r = 0.36, p = 0.02) and non-aneurysm group (r = 0.42, p = 0.006). In addition, significant positive correlations between rupture pressure and length in aneurysm (r = 0.47, p = 0.002) and non-aneurysm groups were noted (r = 0.46, p = 0.002). See Fig. S3A–D online for scatter plots.

**Table 4:** Mean dimensions of specific CAs depending on the presence of unruptured sIA.

	Aneurysm group		Non-aneurysm group		p-value
	Dimension		Dimension		
	Mean	SD	Mean	SD	
ACommA diameter (mm)	2.09	0.60	1.73	0.57	0.24
ACommA length (mm)	2.28	0.39	2.32	0.29	0.82
**ACA**					
A1 diameter (mm)	3.14	0.54	3.39	0.39	0.04
A1 length (mm)	6.72	0.65	6.78	0.75	0.72
A2 diameter (mm)	2.96	0.40	3.18	0.46	0.15
A2 length (mm)	7.64	0.59	6.96	0.61	0.003
ICA diameter (mm)	4.45	0.40	4.67	0.41	0.14
ICA length (mm)	10.03	0.82	10.28	0.70	0.37
**MCA**					
M1 diameter (mm)	3.62	0.33	3.71	0.39	0.32
M1 length (mm)	7.16	0.62	7.07	0.64	0.54
M2 diameter (mm)	2.88	0.44	2.83	0.54	0.68
M2 length (mm)	8.06	0.63	7.79	0.88	0.16
BA diameter (mm)	3.87	0.55	4.20	0.41	0.20
BA length (mm)	9.65	0.54	9.21	0.93	0.26
PCA diameter (mm)	2.73	0.77	2.87	0.62	0.59
PCA length (mm)	8.00	0.67	7.96	0.51	0.83

*ACommA* anterior communicating artery, *ACA* anterior cerebral artery, *A1 and A2* segments of the anterior cerebral artery, *BA* basilar artery, *ICA* internal carotid artery, *MCA* middle cerebral artery, *M1 and M2* segments of the middle cerebral artery, *PCA* posterior cerebral artery, *SD* standard deviation.

### Age dependency of rupture pressure values

**Figure 3:**
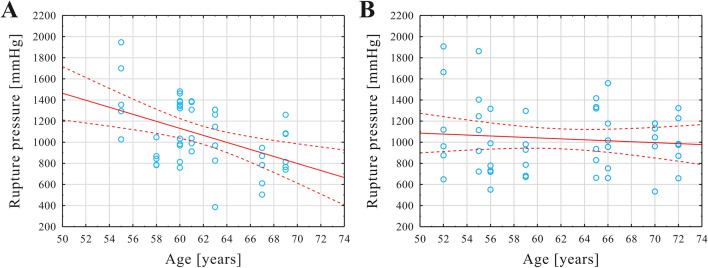
Scatter plot of the rupture pressure values of CAs (shown on y-axis) against age of the autopsy subjects (shown on x-axis) in the aneurysm group (**A**) and non-aneurysm group (**B**). Linear fit is presented as red solid line and its 95% confidence interval (red dotted lines). The position of each light blue open circle indicates values for an individual CA specimen.

No significant differences were noted between the mean age of autopsy subjects in the aneurysm and non-aneurysm groups (p = 0.83). The mean CA rupture pressure in the aneurysm group was negatively correlated with age (r = − 0.45, p = 0.002). In contrast, there was no significant correlation between age and mean CA rupture pressure in the non-aneurysm group (p = 0.508) (Fig. 3A,B).

## Discussion

### *Loci minoris resistentiae* of the cerebral arterial network

In our study, the presence of single unruptured sIA did not influence the rupture pressure values of specific CA segments. Moreover, bifurcation regions displayed high strength, and the rupture pressure did not significantly differ based on location of the rupture of the analyzed CAs, specifically at the wall or bifurcation region. Nevertheless, based on the study of Mitchell and Jakubowski, the average rupture pressure of branched CA specimens was significantly lower than the rupture pressure of straight CA specimens^12^. Alternatively, Ciszek et al. did not observe a significant difference in rupture pressure values between straight and branched CA specimens^13^. In our study, rupture occurred most frequently at the wall of the analyzed CAs. During the pressure-inflation tests, the bifurcation region ruptured in 40% of MCAs from the aneurysm group and in 25% of MCAs and 12.5% of ICAs from the non-aneurysm group. However, such high strength of CA bifurcations may be counter-intuitive when considering that bifurcation regions are histologically characterized by a discontinuity of the media^14^ and enlarged fenestrations within the internal elastic lamina^4^. Finlay et al. indicated that the arrangement of adventitial collagen fibers at the bifurcation apex differs from that in the straight arterial segments. In the bifurcation region, collagen fibers are densely co-aligned and form a strong, tendon-like band that provides high strength of the CA bifurcation^15^.

The sIA development comprises a sequence of overlapping adaptive and degenerative processes induced by specific hemodynamic factors. Only when degenerative processes prevail over adaptive processes does wall strength decrease, leading to sIA rupture^9^. Thus, not all sIAs correspond to regions of low wall strength. In a study by Ciszek et al., one sIA ruptured under pressure markedly exceeding physiological blood pressure, while another sIA sustained high intraluminal pressure values^13^. Additionally, during the experiments conducted by Mitchell and Jakubowski, the sIA specimen was pressurized up to 1.6 bar (1200 mmHg) and the rupture occurred within the wall of adjacent CA^12^. Similarly, in our study, none of the analyzed sIAs ruptured at physiological pressures. Furthermore, in the case of five sIA specimens, the rupture occurred remotely from the sIA, indicating its high wall strength. According to the lifelong Finnish cohort follow-up study, approximately 70% of sIAs did not rupture during the lifetime observation^16^ which is in line with the results of our study, as well as the aforementioned CAs pressure-inflation test studies.

Specific CA segments, especially ACommA, that are prone to sIA formation simultaneously predispose to an increased risk of sIA rupture. In a retrospective epidemiological study by Carter et al., ruptured sIAs were most frequently located in the ACommA complex. Additionally, the mean size of sIAs involving ACommA or distal branches of major CAs was smaller than that of sIAs observed at larger CAs, such as the ICA or MCA bifurcation. Carter et al. hypothesized that there is a decrease in sIA wall thickness along with a decrease in the caliber of the parent artery^17^. It may be reasonable to consider Laplace’s law applied to this context: when assuming similar pressure conditions and the same diameter, sIAs with thin walls experience remarkably higher intramural stress than their thick-wall counterparts. Likewise, in our study, ACommA displayed significantly lower mean rupture pressure than the ICA and MCA bifurcation in both the aneurysm and non-aneurysm groups. Moreover, CAs with smaller diameter and length were characterized by lower rupture pressure values. However, in a study by Ciszek et al., as well as in a study by Mitchell and Jakubowski, the authors did not observe significant differences between rupture pressure values depending on the CA segment location^12,13^. Further investigations, including histological studies with the measurement of wall thickness, are required to clarify how caliber of CAs is related to the strength of their wall.

### Mechanobiology of the CAs

The rupture pressure values of CAs in both analyzed groups markedly exceeded the maximal in vivo arterial blood pressure^18^ which is consistent with the results of earlier pressure-inflation tests of CAs. In a study of Mitchell and Jakubowski, the average rupture pressure of all analyzed CAs was 1.848 bar (1386 mmHg), while the mean CAs rupture pressure presented in a study conducted by Ciszek et al. was 2.35 atm (1786 mmHg)^12,13^. Such discrepancies in the literature data regarding rupture pressure values may result from the diverse age distribution of CAs donors. The mean age at the time of death of autopsy subjects was 69 years in the former study and 47 years in the latter study. Additionally, both studies revealed a significant negative dependency of CA rupture pressure with age. Thus, the relatively higher mean CA rupture pressure obtained by Ciszek et al. may be explained by the predominance of CAs harvested from younger autopsy subjects.

Gradual decrease of the rupture pressure of CAs with age, similar to the gradual increase with age of CA stiffness, may be attributable to age-related arterial wall remodeling, including internal elastic lamina degeneration^5^. Specifically, elastin is the main load-bearing material at low strain conditions, while collagen fibers maintain structural integrity of the CA during further increase of mechanical loadings. Progressive elastin degradation results in an age-related increase in CA stiffness at low strain regimes, indicating early recruitment of collagen fibers. Since collagen fibers are capable of slight deformations prior to failure, once the elastin function is impaired, the intramural stress within the CA wall increases under the same pressure conditions^19^. Nevertheless, in case of composed mainly by collagen, separated CA adventitia specimens, the negative association between the rupture pressure values and age was also observed^20^. This may be explained by the stiffening of collagen fibers during ageing. According to the study of Wuyts et al. the transition strain, i.e. the strain at which collagen fibers are becoming involved in the mechanical response, decreases with age^21^. Therefore, it may be conjectured that a significant decrease in rupture pressure values with age exclusively among CAs in the aneurysm group indicates more pronounced progression of elastin degradation and collagen stiffening within the arterial wall compared to CAs in the non-aneurysm group. However, we did not find a significant difference between the experimental and control groups in terms of mean CA rupture pressure. This discrepancy may be explained by the narrow range of age distribution of CAs donors. If CAs from younger and older autopsy subjects had been pressurized, then CAs from the non-aneurysm group may have presented similar negative dependency of rupture pressure with age.

## Limitations

The comparison of rupture pressure values between sIAs and corresponding CA segments was limited by the small number of ruptured sIAs during the pressure-inflation tests. Diameter and length of the CA specimens were registered only at the beginning of the experiment, so the investigation of the pressure-strain relation was not possible. Also, all the analyzed CA and sIA specimens were obtained from the autopsy subjects and the experiments were conducted within 36 h postmortem. Furthermore, in the case of CAs harvested from human autopsy subjects, the smooth muscles of the wall are consequently without tone. Due to differences between biomechanical properties of CAs collected during surgery and autopsy, presented results may not completely reflect in vivo wall strength of both CAs and sIAs^22^. Alternatively, only in the case of pressure-inflation tests conducted on the specimens harvested from the autopsy subjects, rupture pressure values of sIAs and major CAs may be assessed.

## Conclusions

The mean CA rupture pressure did not significantly differ between the aneurysm and non-aneurysm groups, and markedly exceeded the maximal in vivo arterial blood pressure values. Thus, it may be concluded that the presence of unruptured sIA is not related to the generalized weakening of the CAs. Moreover, CA bifurcations were not identified as regions of decreased wall strength. Alternatively, location of the ACommA of the analyzed CA segment and smaller dimensions of the CA as well as older age of the autopsy subject are associated with lower rupture pressure values. Nevertheless, in our study, a significant negative correlation between CA rupture pressure values and age associated with sIA presence requires further research conducted on CAs donors with a greater age distribution.

## Supplementary Information

Supplementary Information.

Supplementary Figure S1.

Supplementary Figure S2.

Supplementary Figure S3.

## Data Availability

The data that support the findings of this study are available from the corresponding author upon reasonable request.
