# Supplementary material for: Rupture pressure values of cerebral arteries in the presence of unruptured intracranial aneurysm
Source: Sci Rep. 2022 Jun 18;12:10294. doi: 10.1038/s41598-022-13341-8 (PMC9206654; doi:10.1038/s41598-022-13341-8)
Supplement: Supplementary file 1 — Supplementary Information. [file 41598_2022_13341_MOESM1_ESM.docx]

Figure S1.

Exemplary pressure recording obtained during preconditioning cycles and pressure-inflation test. A rupture moment is clearly visible as a sudden drop of pressure.

Figure S2.

Schematic representation of the dimensions of analyzed BA bifurcations, ICA bifurcations, MCA bifurcations (A and B), and ACommA complexes (C and D) measured at intraluminal pressure of 100 mmHg.

Abbreviations: D_1_, D_2_, D_3_ - diameters of the particular arteries; D_a_ - diameter of the ACommA; L_1_, L_2_, L_3_ - lengths of the particular arteries; L_a_ - length of the ACommA.

Figure S3.

Scatter plots of the rupture pressure values (shown on y-axis) against the average diameter of CAs (shown on x-axis) in the aneurysm group (A) and non-aneurysm group (B) as well as the rupture pressure values (shown on y-axis) against the length of CAs (shown on x-axis) in the aneurysm group (C) and non-aneurysm group (D). Linear fit is presented as red solid line and its 95% confidence interval (red dotted lines). The position of each light blue open circle indicates values for an individual CA specimen.

Table S1. Mean rupture pressure of ICA and MCA segments depending on the side (right vs. left) in aneurysm and non-aneurysm group.

| **Side** | | | | | | | | | |
| --- | --- | --- | --- | --- | --- | --- | --- | --- | --- |
|  | **Right** | | | | | **Left** | | |  |
| **Rupture pressure** | | | | | | | | | |
|  | | **Mean** | | **SD** | **Mean** | | **SD** | | **p-value** |
| **Aneurysm group** | | | | | | | | | |
| **ICA (mmHg)** | | 1181 | | 205 | 1340 | | 312 | | 0.26 |
| **MCA (mmHg)** | | 1122 | | 328 | 1112 | | 289 | | 0.95 |
| **Non-aneurysm group** | | | | | | | | | |
| **ICA (mmHg)** | 1127 | | 179 | | | 1166 | | 316 | 0.76 |
| **MCA (mmHg)** | 1021 | | 331 | | | 1190 | | 409 | 0.38 |

Abbreviations: ICA, internal carotid artery; MCA, middle cerebral artery; SD, standard deviation.

Table S2. Rupture pressure values, rupture sites and arterial dimensions of the anterior communicating complexes.

| **Group** | **Age** | **Sex** |  | **Rupture site** | | **Arterial dimensions** | | | | | | | | | |
| --- | --- | --- | --- | --- | --- | --- | --- | --- | --- | --- | --- | --- | --- | --- | --- |
|  |  |  | **Rupture pressure**  **(mmHg)** | **Aneurysm** | **Artery** | **ACommA diameter (mm)** | **ACommA length (mm)** | **L A1 diameter (mm)** | **L A1 length (mm)** | **L A2 diameter (mm)** | **L A2 length (mm)** | **R A1 diameter (mm)** | **R A1 length (mm)** | **R A2 diameter (mm)** | **R A2 length (mm)** |
| Aneurysm | 60 | M | 803 | - | + | 2.31 | 2.48 | 3.21* | 6.46* | 2.96 | 7.13 | 3.45 | 6.53 | 2.67 | 7.77 |
| Aneurysm | 55 | F | 672 | + | - | 1.76 | 2.21 | 2.44 | 6.81 | 2.41 | 8.76 | 2.63 | 6.98 | 2.36 | 7.62 |
| Aneurysm | 60 | M | 749 | - | + | 3.23 | 1.69 | 3.55 | 6.04 | 2.61* | 7.20* | 2.02 | 7.10 | 3.51 | 7.99 |
| Aneurysm | 63 | F | 381 | - | + | 1.83* | 2.17* | 3.01 | 6.14 | 3.39 | 6.85 | 3.56 | 7.81 | 3.48 | 7.66 |
| Aneurysm | 67 | M | 498 | - | + | 1.59* | 2.13* | 2.78 | 5.94 | 2.65 | 7.81 | 2.83 | 6.02 | 2.69 | 6.79 |
| Aneurysm | 58 | M | 858 | - | + | 1.78* | 1.99* | 3.21 | 5.40 | 2.67 | 7.09 | 3.27 | 6.59 | 2.78 | 7.23 |
| Aneurysm | 69 | M | 803 | - | + | 2.69* | 2.91* | 3.83 | 6.35 | 3.26 | 8.68 | 2.10 | 7.50 | 3.24 | 8.15 |
| Aneurysm | 61 | M | 902 | - | + | 1.54* | 2.68* | 3.51 | 7.10 | 3.43 | 7.98 | 3.19 | 6.76 | 3.19 | 7.56 |
| Non-aneurysm | 56 | M | 722 | - | + | 1.5* | 2.65* | 2.72 | 5.12 | 2.76 | 6.34 | 3.46 | 6.22 | 3.42 | 6.71 |
| Non-aneurysm | 59 | M | 663 | - | + | 1.15* | 2.03* | 4.02 | 7.53 | 4.31 | 7.37 | 3.54 | 8.81 | 3.58 | 6.95 |
| Non-aneurysm | 55 | M | 713 | - | + | 2.64* | 1.86* | 3.36 | 5.91 | 3.10 | 5.82 | 3.26 | 6.84 | 3.52 | 6.81 |
| Non-aneurysm | 65 | F | 655 | - | + | 2.53* | 2.46* | 3.62 | 6.02 | 3.21 | 5.97 | 3.33 | 6.43 | 3.15 | 6.67 |
| Non-aneurysm | 72 | F | 650 | - | + | 1,67 | 2,12 | 3,43* | 6,78* | 3,14 | 7,03 | 3,75 | 7,15 | 3,60 | 7,28 |
| Non-aneurysm | 70 | M | 527 | - | + | 1.26* | 2.52* | 3.21 | 6.45 | 3.01 | 7.36 | 3.49 | 6.80 | 3.10 | 7.53 |
| Non-aneurysm | 52 | M | 641 | - | + | 1.32 | 2.58 | 2.69 | 5.01 | 2.65 | 6.99 | 2.49* | 5.91* | 2.31 | 6.85 |
| Non-aneurysm | 66 | M | 653 | - | + | 1.77 | 2.35 | 3.35* | 6.40* | 3.15 | 7.30 | 3.35 | 6.50 | 2.90 | 8.34 |

Abbreviations: +, present; -, absent; M, male; F, female; L, left; R, right; ACommA, anterior communicating artery; A1 and A2, segments of the anterior cerebral artery; * indicates the diameter and length of the artery that ruptured during the experiment.

Table S3. Rupture pressure values, rupture sites and arterial dimensions of the basilar arteries.

| **Group** | **Age** | **Sex** |  | **Rupture site** | | | **Arterial dimensions** | | | | | |
| --- | --- | --- | --- | --- | --- | --- | --- | --- | --- | --- | --- | --- |
|  |  |  | **Rupture pressure**  **(mmHg)** | **Aneurysm** | **Bifurcation** | **Artery** | **BA diameter (mm)** | **BA length (mm)** | **L PCA diameter (mm)** | **L PCA length (mm)** | **R PCA diameter (mm)** | **R PCA length (mm)** |
| Aneurysm | 60 | M | 1021 | - | - | + | 3.54 | 9.76 | 1.47* | 7.60* | 3.40 | 8.63 |
| Aneurysm | 55 | F | 1014 | - | - | + | 3.53 | 10.02 | 2.63 | 8.93 | 2.56* | 8.82* |
| Aneurysm | 60 | M | 971 | - | - | + | 4.06* | 9.76* | 3.31 | 7.56 | 3.56 | 8.85 |
| Aneurysm | 63 | F | 815 | - | - | + | 3.40* | 8.72* | 1.86 | 8.46 | 2.47 | 7.34 |
| Aneurysm | 67 | M | 774 | - | - | + | 3.67 | 8.89 | 2.44* | 6.73* | 2.52 | 7.78 |
| Aneurysm | 58 | M | 776 | - | - | + | 4.69* | 10.14* | 3.30 | 7.46 | 3.26 | 7.61 |
| Aneurysm | 69 | M | 756 | - | - | + | 3.37 | 9.99 | 3.01 | 8.46 | 0.98* | 7.59* |
| Aneurysm | 61 | M | 977 | - | - | + | 4.70* | 9.94* | 3.41 | 7.59 | 3.53 | 8.63 |
| Non-aneurysm | 56 | M | 769 | - | - | + | 4.57 | 8.30 | 3.01 | 7.20 | 3.18* | 7.70* |
| Non-aneurysm | 59 | M | 776 | - | - | + | 4.77* | 9.84* | 3.66 | 8,74 | 3,24 | 7,47 |
| Non-aneurysm | 55 | M | 1385 | - | - | + | 4.13* | 10.13* | 3.62 | 8.10 | 3.40 | 8.73 |
| Non-aneurysm | 65 | F | 819 | - | - | + | 3.37 | 7.68 | 1.49* | 7.87* | 2.39 | 7.45 |
| Non-aneurysm | 72 | F | 860 | - | - | + | 4.17 | 8.41 | 3.04* | 7.27* | 2.98 | 8.15 |
| Non-aneurysm | 70 | M | 949 | - | - | + | 4.02 | 9.81 | 2.46* | 7.93* | 2.50 | 7.98 |
| Non-aneurysm | 52 | M | 1105 | - | - | + | 4.23* | 9.74* | 1.69 | 8.2 | 3.21 | 8.62 |
| Non-aneurysm | 66 | M | 1004 | - | - | + | 4.32* | 9.78* | 2.86 | 8.42 | 3.13 | 7.47 |

Abbreviations: +, present; -, absent; M, male; F, female; L, left; R, right; BA, basilar artery; PCA, posterior cerebral artery; * indicates the diameter and length of the artery that ruptured during the experiment.

Table S4. Rupture pressure values, rupture sites and arterial dimensions of the left internal carotid arteries.

| **Group** | **Age** | **Sex** |  | **Rupture site** | | | **Arterial dimensions** | | | | | |
| --- | --- | --- | --- | --- | --- | --- | --- | --- | --- | --- | --- | --- |
|  |  |  | **Rupture pressure**  **(mmHg)** | **Aneurysm** | **Bifurcation** | **Artery** | **ICA diameter (mm)** | **ICA length (mm)** | **A1 diameter (mm)** | **A1 length (mm)** | **M1 diameter (mm)** | **M1 length (mm)** |
| Aneurysm | 60 | M | 1323 | - | - | + | 4.09* | 10.30* | 3.41 | 7.23 | 3.68 | 6.87 |
| Aneurysm | 55 | F | 1921 | - | - | + | 4.44* | 10.04* | 2.66 | 6.89 | 3.04 | 7.25 |
| Aneurysm | 60 | M | 1372 | - | - | + | 5.30 | 10.91 | 4.05* | 6.79* | 4.25 | 6.34 |
| Aneurysm | 63 | F | 1292 | - | - | + | 4.42* | 10.11* | 3.18 | 5.88 | 3.57 | 7.17 |
| Aneurysm | 67 | M | 859 | - | - | + | 4.52 | 10.88 | 2.94* | 6.43* | 3.33 | 7.25 |
| Aneurysm | 58 | M | 1017 | + | - | - | 4.55 | 8.74 | 3.41 | 7.23 | 3.62 | 6.87 |
| Aneurysm | 69 | M | 1243 | - | - | + | 5.06 | 9.42 | 3.98* | 6.56* | 4.34 | 7.47 |
| Aneurysm | 61 | M | 1370 | - | - | + | 4.19* | 8.55* | 3.74 | 7.44 | 3.87 | 6.98 |
| Non-aneurysm | 56 | M | 977 | - | - | + | 4.94* | 10.24* | 3.22 | 8.37 | 4.41 | 7.52 |
| Non-aneurysm | 59 | M | 1280 | - | - | + | 4.93* | 10.08* | 3.46 | 7.03 | 3.83 | 6.04 |
| Non-aneurysm | 55 | M | 1838 | - | - | + | 4.87* | 10.21* | 3.75 | 7.44 | 3.58 | 6.21 |
| Non-aneurysm | 65 | F | 1303 | - | + | - | 4.34 | 9.98 | 3.71 | 6.59 | 3.45 | 7.14 |
| Non-aneurysm | 72 | F | 963 | - | + | - | 4.61 | 10.64 | 3,65 | 7,23 | 3,94 | 6,91 |
| Non-aneurysm | 70 | M | 1164 | - | - | + | 4.95* | 10.54* | 3.45 | 7.12 | 3.89 | 7.69 |
| Non-aneurysm | 52 | M | 865 | - | - | + | 3.94 | 8.05 | 2.78 | 7.18 | 3.11* | 7.47* |
| Non-aneurysm | 66 | M | 943 | - | - | + | 4.26* | 10.74* | 3.43 | 6.84 | 3.53 | 6.71 |

Abbreviations: +, present; -, absent; M, male; F, female; ICA, internal carotid artery; A1, segment of the anterior cerebral artery; segment of the middle cerebral artery; * indicates the diameter and length of the artery that ruptured during the experiment.

Table S5. Rupture pressure values, rupture sites and arterial dimensions of the right internal carotid arteries.

| **Group** | **Age** | **Sex** |  | **Rupture site** | | | **Arterial dimensions** | | | | | | |
| --- | --- | --- | --- | --- | --- | --- | --- | --- | --- | --- | --- | --- | --- |
|  |  |  | **Rupture pressure**  **(mmHg)** | **Aneurysm** | **Bifurcation** | **Artery** | **ICA diameter (mm)** | **ICA length (mm)** | **A1 diameter (mm)** | **A1 length (mm)** | **M1 diameter (mm)** | | **M1 length (mm)** |
| Aneurysm | 60 | M | 1462 | - | - | + | 4.26* | 11.00* | 3.60 | 7.50 | 3.74 | 8.62 | |
| Aneurysm | 55 | F | 1337 | - | - | + | 4.20 | 9.54 | 2.79* | 7.38* | 3.56 | 7.94 | |
| Aneurysm | 60 | M | 1364 | - | - | + | 4.03 | 9.87 | 2.14* | 5.83* | 3.85 | 7.36 | |
| Aneurysm | 63 | F | 957 | - | - | + | 4.22* | 9.39* | 3.71 | 6.15 | 3.79 | 6.97 | |
| Aneurysm | 67 | M | 934 | - | - | + | 4.34* | 10.92* | 3.07 | 6.32 | 3.51 | 7.11 | |
| Aneurysm | 58 | M | 1033 | - | - | + | 5.10 | 10.72 | 3.45* | 6.73* | 3.89 | 7.24 | |
| Aneurysm | 69 | M | 1070 | - | - | + | 4.09 | 10.49 | 2.34* | 6.86* | 3.26 | 6.92 | |
| Aneurysm | 61 | M | 1292 | - | - | + | 4.20 | 9.17 | 3.39 | 7.15 | 3.92* | 8.28* | |
| Non-aneurysm | 56 | M | 1299 | - | - | + | 5.21* | 10.89* | 4.06 | 6.95 | 4.36 | 8.07 | |
| Non-aneurysm | 59 | M | 917 | - | - | + | 5.06* | 11.02* | 3.39 | 7.18 | 4.12 | 8.19 | |
| Non-aneurysm | 55 | M | 906 | - | - | + | 4.95* | 10.53* | 3.26 | 6.88 | 3.75 | 7.79 | |
| Non-aneurysm | 65 | F | 1316 | - | - | + | 4.51* | 10.12* | 3.65 | 6.72 | 3.45 | 7.59 | |
| Non-aneurysm | 72 | F | 1305 | - | - | + | 4.73 | 10.74 | 3,96 | 6,88 | 4,17* | 7,12* | |
| Non-aneurysm | 70 | M | 1161 | - | - | + | 5.00* | 10.69* | 3.62 | 7.04 | 4.27 | 7.66 | |
| Non-aneurysm | 52 | M | 949 | - | - | + | 3.80* | 9.59* | 2.60 | 6.46 | 3.39 | 6.97 | |
| Non-aneurysm | 66 | M | 1161 | - | - | + | 4.65* | 10.43* | 3.41 | 7.11 | 3.66 | 7.49 | |

Abbreviations: +, present; -, absent; M, male; F, female; ICA, internal carotid artery; A1, segment of the anterior cerebral artery; segment of the middle cerebral artery; * indicates the diameter and length of the artery that ruptured during the experiment.

Table S6. Rupture pressure values, rupture sites and arterial dimensions of the left middle cerebral arteries.

| **Group** | **Age** | **Sex** |  | **Rupture site** | | | **Arterial dimensions** | | | | | |
| --- | --- | --- | --- | --- | --- | --- | --- | --- | --- | --- | --- | --- |
|  |  |  | **Rupture pressure**  **(mmHg)** | **Aneurysm** | **Bifurcation** | **Artery** | **M1 diameter (mm)** | **M1 length (mm)** | **M2 diameter (mm)** | **M2 length (mm)** | **M2’ diameter (mm)** | **M2’ length (mm)** |
| Aneurysm | 60 | M | 956 | - | + | - | 3.67 | 6.10 | 3.56 | 8.80 | 2.83 | 9.04 |
| Aneurysm | 55 | F | 1277 | - | + | - | 2.97 | 7.26 | 2.65 | 8.06 | 2.34 | 7.01 |
| Aneurysm | 60 | M | 1443 | - | - | + | 3.89 | 6.35 | 3.36* | 7.29* | 2.53 | 8.07 |
| Aneurysm | 63 | F | 1245 | - | - | + | 3.45 | 7.37 | 3.34 | 8.66 | 2.31* | 7.62* |
| Aneurysm | 67 | M | 588 | + | - | - | 3.16 | 6.38 | 2.85 | 8.24 | 2.63 | 8.09 |
| Aneurysm | 58 | M | 773 | - | - | + | 3.40 | 6.68 | 3.12* | 7.46* | 1.91 | 8.02 |
| Aneurysm | 69 | M | 730 | - | + | - | 4.14 | 7.26 | 3.53 | 7.81 | 3.22 | 8.40 |
| Aneurysm | 61 | M | 1363 | - | - | + | 3,62 | 7,78 | 3,41* | 8,53* | 2,97 | 6,52 |
| Non-aneurysm | 56 | M | 544 | - | - | + | 4.21 | 7.37 | 2.91 | 7.25 | 2.79* | 6.69* |
| Non-aneurysm | 59 | M | 967 | - | - | + | 3.72 | 7.17 | 3.48 | 9.70 | 1.89* | 10.34* |
| Non-aneurysm | 55 | M | 1100 | - | + | - | 3.47 | 6.58 | 3.39 | 8,72 | 2.16 | 8.18 |
| Non-aneurysm | 65 | F | 1398 | - | - | + | 3.28* | 7.05* | 3.03 | 7.87 | 1.79 | 7.62 |
| Non-aneurysm | 72 | F | 971 | - | - | + | 3.76 | 7.23 | 3.15* | 7.53* | 2.92 | 7.14 |
| Non-aneurysm | 70 | M | 1115 | - | - | + | 3.77 | 6.67 | 3.17* | 7.90* | 2.65 | 8.46 |
| Non-aneurysm | 52 | M | 1883 | - | - | + | 3.21* | 6.88* | 2.5 | 7.99 | 2.09 | 8.67 |
| Non-aneurysm | 66 | M | 1539 | - | + | - | 3.38 | 7.45 | 2.99 | 8.71 | 2.18 | 7.46 |

Abbreviations: +, present; -, absent; M, male; F, female; M1, M2 and M2’, segments of the middle cerebral artery; M2, larger branch; M2’, smaller branch; * indicates the diameter and length of the artery that ruptured during the experiment.

Table S7. Rupture pressure values, rupture sites and arterial dimensions of the right middle cerebral arteries.

| **Group** | **Age** | **Sex** |  | **Rupture site** | | | **Arterial dimensions** | | | | | |
| --- | --- | --- | --- | --- | --- | --- | --- | --- | --- | --- | --- | --- |
|  |  |  | **Rupture pressure**  **(mmHg)** | **Aneurysm** | **Bifurcation** | **Artery** | **M1 diameter (mm)** | **M1 length (mm)** | **M2 diameter (mm)** | **M2 length (mm)** | **M2’ diameter (mm)** | **M2’ length (mm)** |
| Aneurysm | 60 | M | 1304 | - | - | + | 3.68 | 8.06 | 2.91 | 8.25 | 2.59* | 8.93* |
| Aneurysm | 55 | F | 1678 | - | - | + | 3.44 | 5.68 | 2.84 | 8.51 | 2.41* | 8.96* |
| Aneurysm | 60 | M | 1337 | - | + | - | 3.74 | 7.06 | 3.37 | 7.69 | 2.61 | 8.28 |
| Aneurysm | 63 | F | 1132 | - | - | + | 3.64* | 6.78* | 2.92 | 7.16 | 2.53 | 8.73 |
| Aneurysm | 67 | M | 603 | - | - | + | 3.37* | 7.91* | 3.05 | 7.71 | 2.99 | 8.63 |
| Aneurysm | 58 | M | 833 | - | - | + | 3,74 | 7,05 | 3.57* | 7.98* | 2.42 | 6.93 |
| Aneurysm | 69 | M | 1065 | - | + | - | 3.07 | 7.45 | 2.98 | 8.18 | 2.32 | 8.48 |
| Aneurysm | 61 | M | 1025 | - | + | - | 3.76 | 7.45 | 3.54 | 8.24 | 2.46 | 7.62 |
| Non-aneurysm | 56 | M | 710 | - | - | + | 3.97 | 7.73 | 2.65 | 6.77 | 2.38* | 6.32* |
| Non-aneurysm | 59 | M | 674 | - | + | - | 3.68 | 5.68 | 3.36 | 7.23 | 2.51 | 7.12 |
| Non-aneurysm | 55 | M | 1229 | - | - | + | 3.68 | 5.59 | 3.27 | 8.81 | 2.94* | 7.54* |
| Non-aneurysm | 65 | F | 925 | - | + | - | 3.41 | 6.23 | 3,39 | 7.36 | 2.21 | 6.43 |
| Non-aneurysm | 72 | F | 1211 | - | - | + | 4.09* | 7.38* | 3.77 | 7.98 | 3,34 | 7,57 |
| Non-aneurysm | 70 | M | 1033 | - | - | + | 4.15 | 6.41 | 3.60 | 8.07 | 3.48* | 7.73* |
| Non-aneurysm | 52 | M | 1642 | - | - | + | 3.03 | 7.07 | 2.85 | 8.46 | 1.92* | 7.18* |
| Non-aneurysm | 66 | M | 743 | - | - | + | 3.42 | 7.06 | 2.91* | 7.14* | 2.74 | 7.3 |

Abbreviations: +, present; -, absent; M, male; F, female; M1, M2 and M2’, segments of the middle cerebral artery; M2, larger branch; M2’, smaller branch; * indicates the diameter and length of the artery that ruptured during the experiment.
