# Supplementary figures and images for: Rupture pressure values of cerebral arteries in the presence of unruptured intracranial aneurysm
Source: Sci Rep. 2022 Jun 18;12:10294. doi: 10.1038/s41598-022-13341-8 (PMC9206654; doi:10.1038/s41598-022-13341-8)

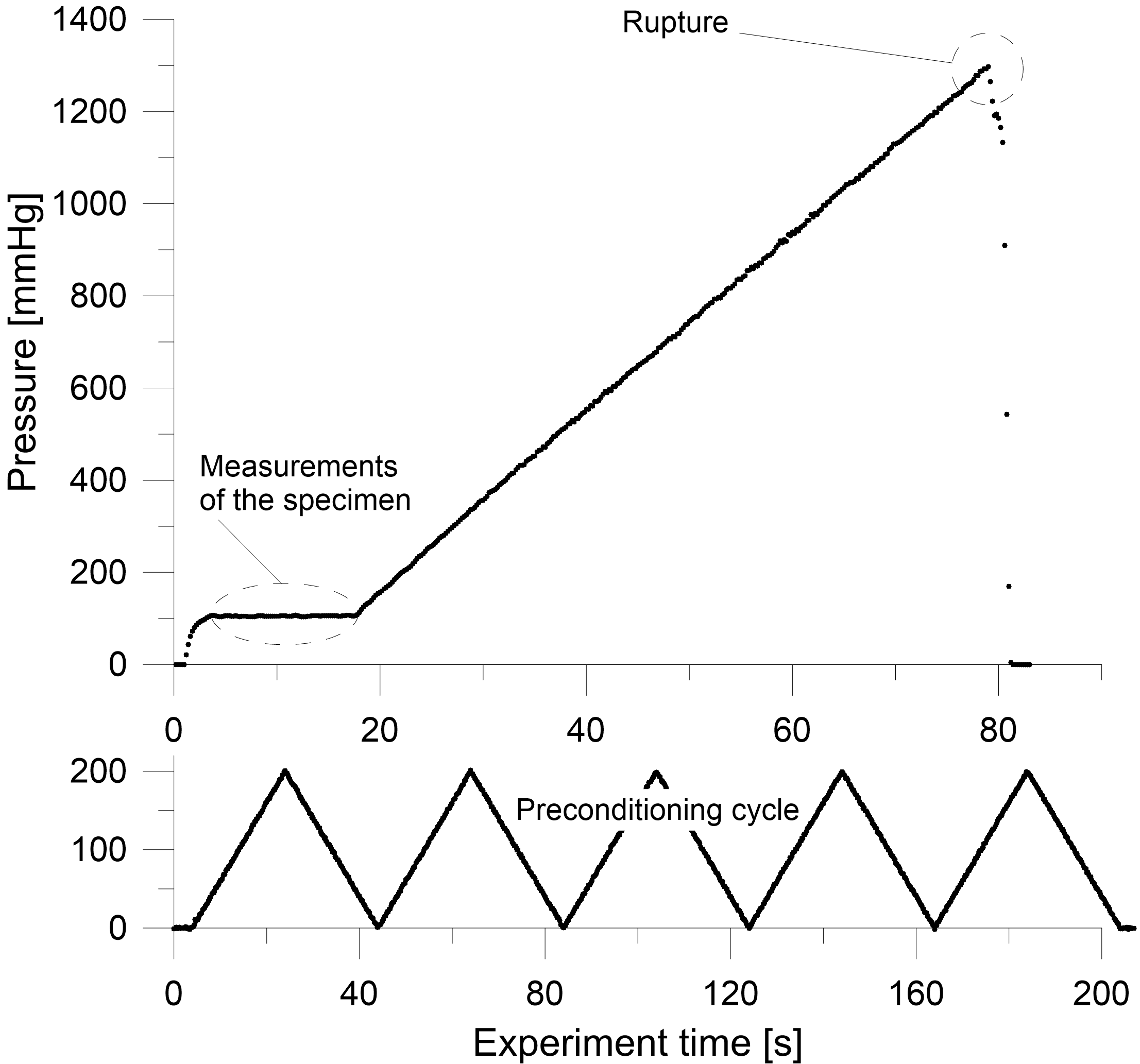

Supplement: Supplementary file 3 — Supplementary Figure S2. [file 41598_2022_13341_MOESM3_ESM.tif]
